# EBM BLS: Treating Anal High-grade Squamous Intraepithelial Lesions Reduces Progression to Invasive Anal Cancer in People Living with HIV

**DOI:** 10.1007/s11606-025-09828-5

**Published:** 2025-09-15

**Authors:** Steven Allon, Jason T. Alexander, Christopher Terndrup

**Affiliations:** 1 Division of General Internal Medicine, Department of Medicine, Vanderbilt University Medical Center, 719 Thompson Lane, Suite 20400, Nashville, TN 37204 USA; 2 University of Chicago, Chicago, IL USA

**Keywords:** anal cancer, screening for cancer, HIV

## WHY THIS IS IMORTANT

- Anal cancer is uncommon in the general population; however, the lifetime risk of anal cancer in people living with HIV (PLWH) is approximately 4%, comparable to that of colorectal cancer.^1^
- The natural history of anal cancer parallels cervical cancer, with > 90% of cases associated with local infection by cancer-causing strains of human papillomavirus (HPV).^2^
- There is a lack of evidence and consistent recommendations about screening and treatment for anal high-grade squamous intraepithelial lesions (HSIL) in high-incidence populations to reduce the risk of progression to anal cancer.^3^
- This is the first large-scale randomized controlled trial to evaluate the efficacy and safety of screening and treating anal HSIL to prevent anal cancer in PLWH.

## INTERVENTION

**Figure 1:**
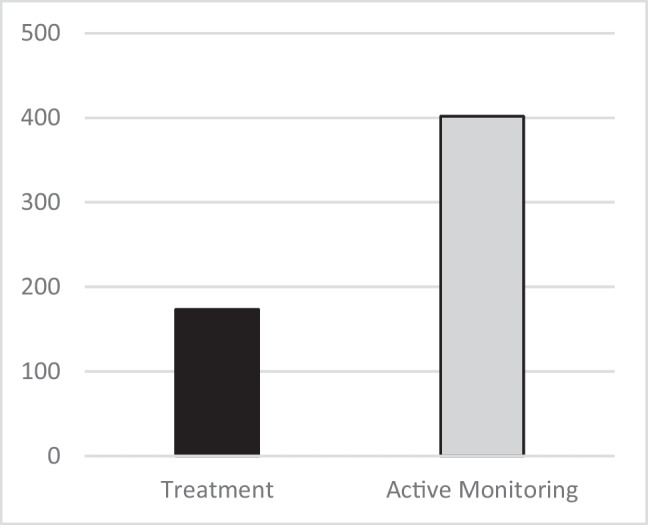
Anal cancer incidence per 100,000 person-years.

- 10,723 participants were screened for trial enrollment, 9,831 participants completed baseline anal swab and high-resolution anoscopy (HRA), and 54.0% were diagnosed with anal HSIL.
- A total of 4,459 participants with HSIL underwent 1:1 randomization to either treatment or active monitoring Fig. 1.

## RESULTS

- Median age was 51 years old, 80% identified as male gender, 42% were Black, and 32% were white.
- Median time from HIV diagnosis was 17 years, and > 80% had an undetectable viral load.
- Anal HSIL was treated in 99.6% of participants in the treatment arm. Most (83.7%) received office-based electrocautery ablation.
- The median follow-up was 25.8 months.
- Anal cancer incidence was 173 per 100,000 person-years in the treatment arm (95% CI 90 to 332) and 402 per 100,000 person-years in the active monitoring arm (95% CI 262 to 616), a 57% reduction (95% CI, 6–80%; p = 0.03).
- Severe trial-related adverse events occurred in 7 participants in the treatment arm and 1 in the active monitoring arm; the most common was postprocedural infection or abscess.

### Study Description

#### Setting

- This study enrolled PLWH aged 35 years or older in the USA between 2014 and 2021.
- Inclusion criteria: biopsy-confirmed anal HSIL (anal intraepithelial neoplasia 3 or 2 with HPV 16 positivity) detected via baseline anal swab and HRA.
- Exclusion criteria: history of anal cancer, or anal cancer detected during screening procedures. HPV vaccination and prior treatment of HSIL were initially exclusion criteria but were removed mid-trial with a protocol modification.

#### Methods

- Patients were randomized in a 1:1 ratio to immediate treatment of anal HSIL or active monitoring of lesions.
- Treatment of anal HSIL followed protocol-defined options, including ablative, excisional, and topical modalities. In the treatment arm, HSIL was treated until resolution, with recurrences managed with additional treatment.
- Both groups underwent surveillance HRA twice yearly with annual biopsy of visible lesions.
- The primary outcome was progression to invasive anal cancer.

#### Study Quality and Application to Patients

- The study quality is good. Its strengths include randomization, adherence, and low dropout rates.
- It addresses a real-world question where practice varies due to a lack of trial evidence.
- The unblinded study design may have impacted biopsy decisions by proceduralists.
- This study serves as a proof-of-concept. However, caution is advised applying the findings to populations with a lower incidence of anal cancer.
- The study was not powered to evaluate the effect of screening on mortality from anal cancer, the standard endpoint for cancer screening trials.
- The high prevalence of anal HSIL presents challenges for healthcare systems that already face a shortage of proceduralists skilled in HRA.
- Mid-trial changes led to inclusion of participants with prior treated anal HSIL (~ 10%) and HPV vaccination (~ 0.5%); the former group may have increased risk of anal cancer, and the latter likely had minimal impact.

#### Tips for Patients

- Screening and treating anal HSIL reduces the risk of invasive anal cancer in people living with HIV.
- No guidelines have been issued by major cancer screening organizations, necessitating shared decision-making between providers and patients.

## Declarations:

### Prior Presentations:

This work has not been previously presented at any conferences.
